# Prediction of *Leishmania major* Key Proteins Via Topological Analysis of Protein-Protein Interaction Network

**DOI:** 10.22086/gmj.v0i0.1129

**Published:** 2018-06-12

**Authors:** Nasrin Amiri Dashatan, Mostafa Rezaie Tavirani, Hakimeh Zali, Mehdi Koushki, Nayebali Ahmadi

**Affiliations:** ^1^Proteomics Research Center, Shahid Beheshti University of Medical Sciences, Tehran, Iran; ^2^Advanced Technologies in Medicine. Shahid Beheshti University of Medical Sciences, Tehran, Iran; ^3^Department of Clinical Biochemistry, Faculty of Medicine, Tehran University of Medical Sciences, Tehran, Iran

**Keywords:** Protein Interaction Networks, Leishmaniasis, *leishmania major*, Gene Ontology, Centrality Analysis

## Abstract

**Background:**

Although leishmaniasis is regarded as a public health problem, no effective vaccine or decisive treatment has been introduced for this disease. Therefore, representing novel therapeutic proteins is essential. Protein-protein Interaction network analysis is a suitable tool to discover novel drug targets for *leishmania major*. To this aim, gene and protein expression data is used for instructing protein network and the key proteins are highlighted.

**Materials and Methods:**

In this computational and bioinformatics study, the protein/gene expression data related to *leishmania major* were studied, and 252 candidate proteins were extracted. Then, the protein networks of these proteins were explored and visualized by using String database and Cytoscape software. Finally, clustering and gene ontology were performed by MCODE and PANTHER databases, respectively.

**Results:**

Based on gene ontology analysis, most of the *leishmania major* proteins were located in cell compartments and membrane. Catalytic activity and binding were regarded as the relevant molecular functions and metabolic and cellular processes were the significant biological process. In this network analysis, UB-EP52, EF-2, chaperonin, Hsp70.4, Hsp60, tubulin alpha and beta chain, and ENOL and LACK were introduced as hub-bottleneck proteins. Based on clustering analysis, Lmjf.32.3270, ENOL and Lmjf.13.0290 were determined as seed proteins in each cluster.

**Conclusion:**

The results indicated that hub proteins play a significant role in pathogenesis and life cycle of *leishmania major*. Further studies of hubs will provide a better understanding of leishmaniasis mechanisms. Finally, these key hub proteins could be a suitable and helpful potential for drug targets and treating leishmaniasis by considering their validation.

## Introduction

*Lishmania*, as a protozoan parasite, is considered as the etiological agent of leishmaniasis, which is responsible for a spectrum of disease including cutaneous lesions, disfiguring mucocutaneous, and fetal visceral infection among 98 tropical and subtropical countries such as Iran. About 350 million people worldwide are at risk of leishmaniasis and more than 12 million people have been affected by different *leishmania* species [1, 2]. *Leishmania* parasites including a dimorphic life-cycle are transmitted by sand-fly vector. Promastigote forms in alimentary tract of sand-fly are extracellularly flagellated and the intracellular amastigotes survive in the macrophages of the mammalian host. The implementation of proteomic technologies is helpful for studying biology, host-pathogen interaction, drug resistance, virulence and therapeutic targets in *leishmania* parasites [3, 4].

Regarding the role of proteins in cellular systems in interactive state, as well as the significance of physical interactions between proteins in most biological processes, computational approaches such as analyzing protein-protein interaction (PPI) networks have been proposed to identify or predict the essential genes and novel drug targets *in silico*, which can help economize the time and cost [5-8]. Protein interaction networks present gene products, which physically interact with each other in cellular systems. PPI network is usually represented as graph where the proteins are represented as the nodes and interactions between proteins are displayed as the edges. Analyzing the topological parameters related to

PPI networks such as degree and betweenness centrality is useful in recognizing the essential proteins in networks and these important proteins can be regarded as the potential drug targets. The number of edge incident on a node is known as “degree”[9]. High degree nodes (hub proteins) are essential for networks about three times more than non-hub proteins. The removal of highly connected proteins in networks is lethal and these nodes are regarded as interesting drug targets in different diseases. Betweenness centrality is another important property of protein- protein interaction networks which is defined as the ratio of the number of shortest paths passing through a node to the total number of paths passing through the nodes [10]. Nodes with high betweenness centrality in PPI networks are called “bottlenecks”. Several studies found that bottlenecks are the key connectors in protein networks and the topological analysis of protein networks can be considered as the essential proteins. As no vaccine is available, the only method for controlling this disease, especially in developing countries, is chemotherapy with pentavalent antimony compounds including Pantostam and Glucantime [11]. Severe side effect and resistance to these drugs is considered as a major clinical problem and the incidence of drug-resistant *leishmania* spp is increasing in several regions [12]. Thus, identifying and developing several new key/significant proteins as potential drug targets play a pivotal role in preventing this problem. These key/significant proteins can be identified by using modern proteomics and bioinformatics approaches for different diseases [13-15] including cutaneous leishmaniasis caused by *L. major*. The present study aims to analyze some topological properties including degree and betweenness centrality related to protein- protein interaction networks of *L. major* in order to identify hub-bottleneck proteins which can be recommended for new potential drug targets in this parasite.

## Materials and Methods

### Type of Study

Analysis of different diseases has attracted attention of researchers and scientists in the biology and medicine fields. Some tropical diseases are targeted by computational and bioinformatics approaches [16, 17]. The present study is a bioinformatics study that used proteomic data of previous studies to analyze the *L. major* protein interaction network.

### Data Collection

During the recent years, an increasing number of studies have been conducted in genomics and proteomics related to leishmaniasis. Thus, in this study, a total of 252 *L. major* proteins/genes were extracted from five studies which investigated genome and proteome of *L. major* during the last year [18-22]. The accession number of the extracted proteins was searched from Uniprot database (http://uniprot.org), as a publicly available web-based tool.

### Functional Classification of Data Set

**Figure-1:**
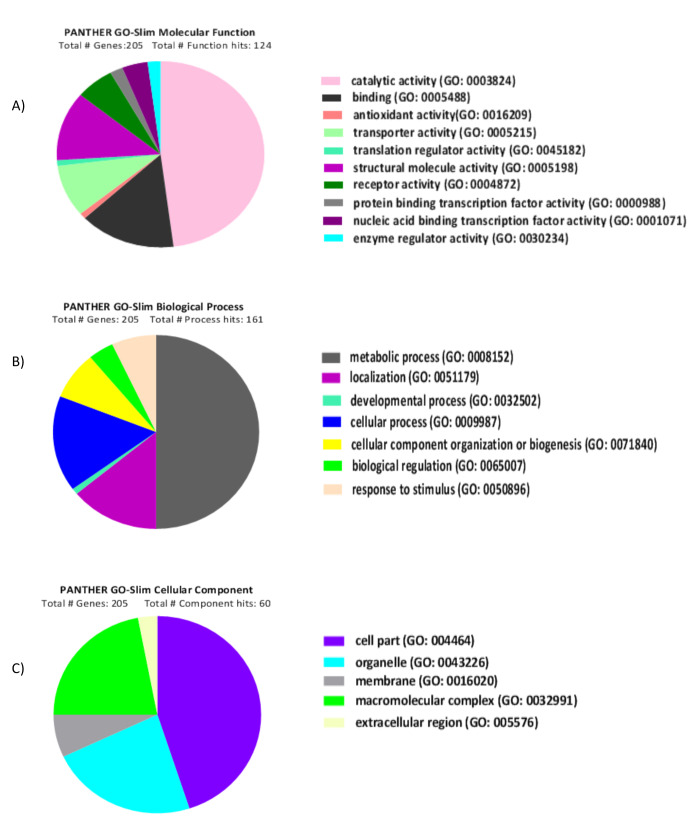
Results of GO Enrichment Analysis Related to *leishmania major* were Performed by PANTHER database. a) Molecular function, b) Biological process and c) Cellular component)

In the present study, the PANTHER (Protein Analysis through Evolutionary Relationships) Classification System which is freely available at http://www.pantherdb.org was used to classify the proteins, as well as their genes. PANTHER tool is a widely used online resource for a comprehensive protein evolutionary and functional classification for large-scale biological data analysis [23]. In this system, after uploading the protein IDs list, *L. major* organism was selected. Finally, the functional classification viewed in pie chart was selected for analysis. Figure-1 illustrates the molecular function, biological process, and cellular component classification of the data set.

### Protein- Protein Interaction Network Analysis

Many molecular processes within a cell are conducted by molecular mechanisms which are built from a large number of protein compounds organized by their interactions. Protein-protein interactions are regarded as the basic principle in self-organization and homeostasis of organisms. In this study, protein IDs was used for constructing protein- protein interaction network in STRING database (http://string-db.org) [24]. First, the disconnected nodes were removed and the protein-protein interaction network was visualized by using the Cytoscape software version 3.0.2 [25]. Then, the protein network was analyzed based on the topological parameters such as degree and betweenness centrality through using Network Analyzer tool in Cytoscape software in order to detect the essential proteins in *L. major* life-cycle. High degree and high betweennesss centrality nodes as the important proteins in *L. major* survival are regarded as the druggable targets for developing new drugs in controlling the parasite. Of course, these putative targets should be specific for *leishmania* parasite. First, the protein network was clustered in order to detect complexes and sub-networks by using Clusterviz application in cytoscape software. In this application, clusters (highly interconnected regions, protein complexes or functional module) were identified by Molecular Complex Detection (MCODE) algorithm to analyze the characteristics of the networks. Interactomes with a score greater than 2.0 and at least two nodes were selected as the significant prediction. Then, the seed nodes as a complex with the highest weighted vertex were identified in each cluster. In the next stage, gene ontology categories were conducted in order to identify in highly connected regions which was generated by MCODE. To this aim, PANTHER (http://pantherdb.org) was used for this classification. PANTHER is a comprehensive system which combines gene function, ontology and pathways. By running the overrepresentation test, the provided list was compared with a reference list of proteins in the PANTHER database. Finally, over- and underrepresentation of ontology categories were statistically determined by Binomial distribution test.

## Results

### Results of Functional Classification of Data Set

First, the gene ontology including biological process, molecular function and cellular component was performed on the list. Bonferroni correction was used for the obtained results. Figure-1 illustrates the molecular function related to *L. major* proteins. As shown in Figure-1a, the catalytic activity is a significant function in data set which is matched with what occurred across *leishmania* infection in host cells. As for Figure-1b, metabolic and cellular process allocated the most percentage in Panther biological process. Based on the results in Figure-1c, cell part is regarded as a significant cellular component related to *L. major* proteins.

### Results of PPIN Analysis

**Figure-2:**
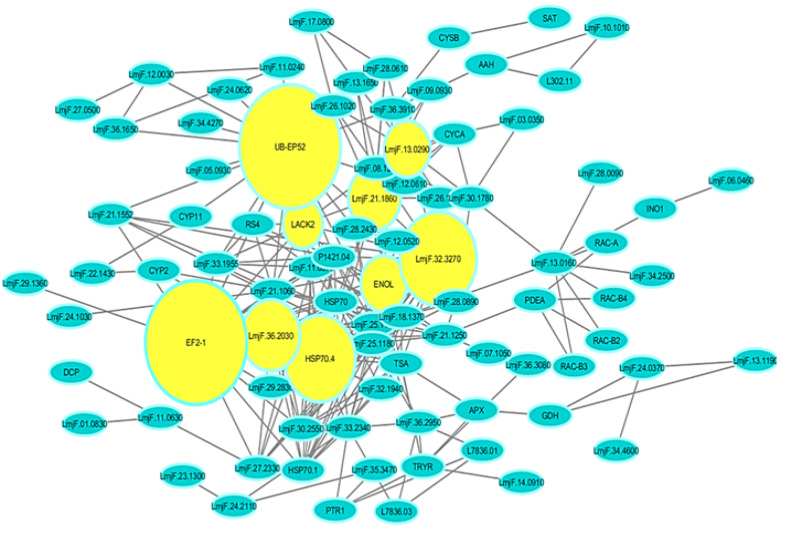
PPI network of *leishmania major* Based on String Database and Visualized by Cytoscape 3.0.2 software. After Visualization of Network, Disconnected Nodes Removed. Hub Nodes With High Degree and Betweenness Centrality Value Shows as Large and Yellow Color Nodes

The protein networks were visualized and analyzed by Cytoscape software after construction by using string database. Then, PPI network including 88 nodes and 265 edges were created after removing the disconnected nodes. Figure-2 illustrates the whole network. Nodes represent the proteins from the list and others which directly interact with them and edges represent physical or functional interaction between the two proteins. The centrality analysis of network was done by using Network Analyzer tool in Cytoscape software. Based on the scale-free network, the node degree distribution is heterogeneous and a majority of nodes include a low node degree. The same rule was implemented in the protein network used in the present study [26]. The betweenness centrality is usually ranged between 0.0 to 1.0 values.

**Figure-3:**
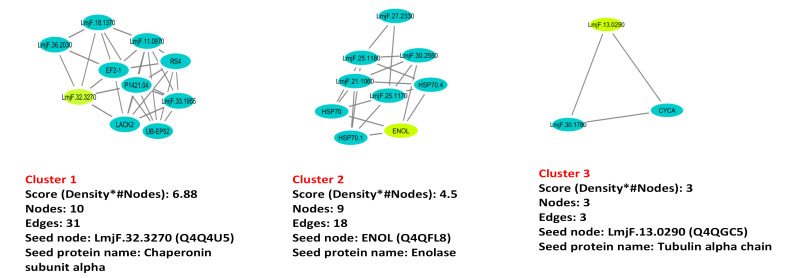
The PPI Subnetworks were Clustered as Highly Connected Regions in leishmania Major Protein-Protein Interaction Network by Clusterviz (MCODE algorithm) Tool in Cytoscape. Yellow Color nodes are Seed Nodes With Highest Weight

**Figure-4:**
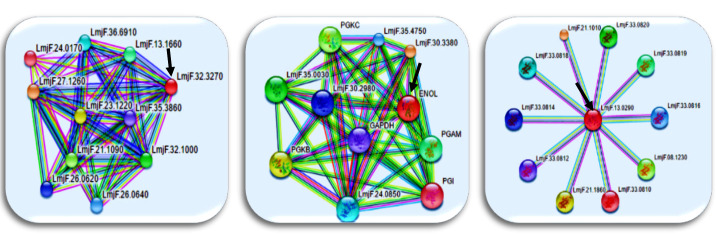
Predicted Interactions for Hub-Seed Proteins were Obtained from STRING Database (http://string-db.org). The Arrows Represent the Hub-eed Proteins (the red colored nodes)

High connectivity and betweenness centrality values indicate the essentiality of proteins in the studied organisms and these proteins could be considered as the potential targets for new drug development. Table-1 represents a number of 9 top essential proteins (hub-bottleneck) with high topologic values (node degree and betweenness centrality). The analysis of small subnetwork by MCODE indicated three clusters for the network. Figure-3 illustrates ranking the subnetworks based on their size. First, the seed nodes were identified in each cluster. Seed nodes are the highest weighted nodes in a graph. The seed nodes of these complexes included Q4Q4U5, Q4QFL8 and Q4QGC5. In addition, the gene ontology analyses for all of the subnetworks generated by MCODE algorithm were performed by PANTHER database (Table-2). The evaluation of protein network interactions represents some valuable information about its role in the systematic function of protein network. STRING resource is regarded as a useful tool for representing these interactions. Figure-4 illustrates the possible interactions for proteins which are either hub-bottleneck and seed nodes due to their importance by using STRING.

**Table-1:** The Hub-bottleneck Proteins With Significant Centrality Values Related to the PPI Network of *L. major* are Presented. ID, D and BC are Abbreviations of Uniprot ID, Degree and Betweenness Centrality Rrespectively

**ID**	**Gene name**	**Protein name**	**D**	**BC**
**P69201**	UB-EP52	Ubiquitin-60S ribosomal protein L40	22	0.24
**Q4Q259**	EF2-2	Elongation factor 2	22	0.08
**Q4Q4U5**	LMJF_32_3270	Chaperonin subunit alpha	21	0.07
**P12077**	HSP70.4	Heat shock 70- related protein 4	16	0.05
**Q4QBZ6**	LMJF_21_1860	tubulin beta chain	14	0.08
**Q4Q1M0**	LMJF_36_2030	Chaperonin Hsp60, mitochondrial	14	0.01
**Q4QFL8**	ENOL	Enolase	13	0.10
**Q4QGC5**	LMJF_13_0280	tubulin alpha chain	10	0.02
**Q4Q7Y7**	LACK2	Activated protein kinase c receptor	10	0.02

**Table-2:** Biological Process Enrichment of Clusters by PANTHER

**PANTHER GO**	***L. major*** ** (REF)#**	** #**	**Expected**	**+/ -**	**p-value**
**protein metabolic process**	801	10	1.25	+	2.13E-06
**Mitosis**	175	3	0.06	+	1.36E-03
**Cell Cycle**	340	3	0.12	+	9.95E-03
**Proteolysis**	184	4	0.29	+	2.12E-02
**Purine nucleobase metabolic process**	35	2	0.03	+	3.84E-02

+/- Shows over- and under representations. Second and third column contains the number of genes in reference list and our list respectively. P-value threshold is considered 0.05. *L: leishmanial*

## Discussion

Molecular mapping of disease, along with their cause, is considered as an effective instrumentation for evaluating molecular pathways [27]. Due to high rate of diverse high-throughput biological data including genomics and proteomics, computational methods have become increasingly successful in inferring protein–protein interactions [28]. Generally, networks analysis is a useful tool for drug target discovering process. Any type of the associated data which is responsible for creating the relationship between one gene, protein and others can be modeled, visualized and analyzed as networks and protein-protein interaction maps can provide a valuable framework for a better understanding of the functional organization of the proteome [27]. Thus, in the present study, PPI network of 252 candidate proteins related to *L. major* is constructed and assessed to predict key proteins as potential drug targets in this disease. Here the proteins are evaluated based on the importance of their role in the network.

One of the advantages of network analysis is the discrimination of a few nodes among too many nodes of a PPI network. As represented in table-1; the numbers of 9 crucial nodes (hub- bottlenecks) which mostly interact with the other nodes are introduced. These proteins were introduced as the essential elements in PPI network of *L. major* and also play important roles in pathophysiology of leishmaniasis.

In this study, UB-EP52 is a hub-bottleneck protein of highest degree and betweenness centrality value, which is considered as the Ubiquitin-60S ribosomal subunit. Ubiquitin is usually conjugated to Lys residues of the target proteins, although conjugation to cysteine or serine residues has been observed in a small number of cases. Polyubiquitin has distinct roles in activating protein kinases and signaling when it is free. Elongation factor 2 is the next hub-bottleneck protein in *L. major* related protein interaction network. Through proteomic technology, elongation factor-2 soluble protein was recognized as a potent immunostimulatory protein which persuades a Th1 response in the PBMCs of the *leishmania* infected patients [29].

Chaperonin subunit alpha, Heat shock 70- related protein 4, Chaperonin Hsp60 are considered as other hub proteins in *L. major* network analysis by centrality parameters. Molecular chaperones play an important role in preserving cellular homeostasis and survival during normal and stress conditions [30]. Based on some documents, chaperones are the key molecules in the life cycle of a vast diversity of human pathogens [31].

Among chaperones, heat shock proteins play a significant role in adapting and surviving *leishmania* parasite when an increase occurs in the temperature during differentiation process [30]. This family of proteins is permanently synthesized during promastigote and amastigote stages which increase during amastigote differentiation, due to parasite response to the temperature stress [32, 33]. Plenty of chaperones have been shown to evoke immune response against *leishmania* infection which is considered as potential vaccine candidates [34]. Hsp70.4 is one of Hsp70 family member without any clear relation among humans [30].

Therefore, since the absence of potential drug target among humans is an important feature in discovering druggable targets, this protein can be selected as the preferred candidate. It is worth noting that there is a relationship between parasitic large Hsps and their virulence potential [35].

In addition, the study of Hsp70.4 localization in *L. major* by immunofluorescence technique indicated that this protein is cytoplasmic even during stress condition. Enolase is another hub-bottleneck protein in present analysis and is described as a key enzyme in glycolysis and glyconeogenesis as two important cellular pathways. Glycolysis play important roles in ATP supply and glyconeogenesis is crucial for the virulence and viability of *leishmania* parasite.

Therefore, these metabolic pathways are regarded as potential targets for antileishmania drugs. Further, it can regulate cell morphology and vesicle trafficking by cytoskeleton system. Furthermore, enolase enzyme is available in secrotome and leishmanial parasite surface. Based on the surface enolase, plasminogen receptor can probably play a role in virulence and invasiveness of parasites [36].

These findings suggest new outlook about vaccine production and drug discovery process. *L. major*. LACK (leishmanial homolog of receptors for activated C-kinase) protein is another hub-bottleneck protein. However, the molecular function of LACK protein is not obvious in *leishmania*. Further, the results of some studies demonstrated an immune response to LACK protein, which is used as an experimental vaccine in animal model in 2005 [37].

LACK antigen in *L. major* is an important target for immunological response among the infected mice and is presented as a possible vaccine candidate in leishmaniasis. Kelly *et al*. (2003) demonstrated that *L. major* with LACK mutant and a decreasing level of this protein failed to develop lesion in susceptible mouse and reduced valence to proliferation in vitro among macrophage cells [38]. Therefore, LACK is critical for the viability of the *leishmania* parasite, which can instate the parasite in the host. Accordingly, LACK plays an important role as a potential drug target, as well as *leishmania* parasite and host interaction. Based on the results, controlling this factor is an effective solution in leishmaniasis. Regarding this study, tubulin-beta chain and tubulin-alpha chain were identified as hub-bottleneck proteins in life cycle of *L. major*. Microtubules associated proteins are involved in the morphology of leishmanial during metacyclogenesis and motility of parasite, especially in metacyclic stage in which this feature of parasite can be important in infective capacity of *leishmania* [20].

In another study on tubulins as an anti-leishmania drug target on L. donvani, tubulins were validated as suitable targets for anti-leishmania drugs [39].

Thus, inhibiting or regulating these hub-bottleneck proteins can considerably influence pathology and control leishmaniasis since these proteins play an important role in the structure of protein network. Furthermore, hub-bottleneck proteins can be critical in biology and parasite survival. Cluster analysis of biological networks is one of the most important approaches for identifying functional modules and predicting protein functions. In addition, the results of clustering visualizationis crucial for uncovering the structure of biological networks. Further, detecting protein complexes is essential for identifying cellular function.

In the present study, protein complexes were determined by MCODE plugin in cytoscape software. The study of the complex through analyzing network with MCODE revealed three subnetworks as described in figure-3. Further, subnetworks were ranked based on their size and were analyzed biological process according to PANTHER GO (Table-2). Furthermore, seed nodes were identified in each subnetwork, as the nodes of heavy weight. Based on the results in the present study, three proteins of chaperonin subunit alpha, enolase and tubulin alpha chain were determined as both hub-bottleneck and seed nodes which can serve as candidate drug targets for leishmaniasis.

But this study is a computational and bioinformatics study and has limitations in using the results of these studies in the clinic. So, for detailed evaluation of these proteins, further experimental investigations and specific inhibitor designs for hub-bottleneck proteins are required.

## Conclusion

Many human diseases are the result of malformed protein– protein interactions involving endogenous proteins, proteins from pathogens or both. The inhibition of these aberrant interactions is of obvious clinical significance. leishmaniasis is one of the most diverse and complex diseases worldwide. In addition, proteomics technology and bioinformatics research has attracted a lot of attention in parasitology fields such as *leishmania* parasite. Integrating network analysis with proteomic methods can help to predict new drug targets and treatments for the disease. This work organizes the first effort to protein interaction networks construction in the *Leishmania major* parasite by utilizing identified proteins/genes by genomics and proteomics methods in previous studies.

We have provided a list of 9 essential proteins to explore as novel potential drug targets.

In the present study, network and GO analysis by using related software indicated that the obtained results can be useful in identifying the relationship between proteins and clarifying the disease mechanism better.

The regulation of the identified hub proteins such as UB-EP52, EF-2, chaperonin, Hsp70.4, Hsp60, tubulin alpha and beta chain, ENOL and LACK can result in controlling the disease and also this information suggests a strong possibility to design drug through these hubs. However, further research is needed for a detailed currency of hubs. In summary, our expanding knowledge of protein–protein interactions analysis at a molecular level is providing great insight into mechanisms of human diseases such as parasitic infections. When combined with recent advances in inhibitor design, this modern field is allowing us to begin to combat a range of infectious diseases.

## Conflict of interest

The authors declared no conflict of interest
